# Combined Effects of Wildfire and Vegetation Cover Type on Volcanic Soil (Functions and Properties) in a Mediterranean Region: Comparison of Two Soil Quality Indices

**DOI:** 10.3390/ijerph18115926

**Published:** 2021-05-31

**Authors:** Lucia Santorufo, Valeria Memoli, Speranza Claudia Panico, Giorgia Santini, Rossella Barile, Antonella Giarra, Gabriella Di Natale, Marco Trifuoggi, Anna De Marco, Giulia Maisto

**Affiliations:** 1 Dipartimento di Biologia, Università degli Studi di Napoli Federico II, Via Cinthia, 80126 Napoli, Italy; lucia.santorufo@unina.it (L.S.); speranzaclaudia.panico@unina.it (S.C.P.); giorgia.santini@unina.it (G.S.); g.maisto@unina.it (G.M.); 2 BAT Center—Interuniversity Center for Studies on Bioinspired Agro-Environmental Technology, University of Naples Federico II, 80126 Naples, Italy; ademarco@unina.it; 3 Parco Nazionale del Vesuvio, Via Palazzo del Principe c/o Castello Mediceo, 80044 Ottaviano, Italy; rbarile@epnv.it; 4 Dipartimento di Scienze Chimiche, Università degli Studi di Napoli Federico II, Via Cinthia, 80126 Napoli, Italy; antonella.giarra@unina.it (A.G.); gabriella.dinatale@unina.it (G.D.N.); marco.trifuoggi@unina.it (M.T.); 5 CeSMA-Centro Servizi Metrologici e Tecnologici Avanzati, Università degli Studi di Napoli Federico II, Corso Nicolangelo Protopisani, 80146 San Giovanni a Teduccio, Italy; 6 Dipartimento di Farmacia, Università degli Studi di Napoli Federico II, Via Montesano, 80131 Napoli, Italy

**Keywords:** volcanic soil, quality index, soil characteristics, plant diversity, fire impact, ecosystem functions

## Abstract

Mediterranean regions are the most impacted by fire in Europe. The effects of fire on soil greatly vary according to several factors such as vegetation cover type, but they are scarcely studied. Therefore, this research aimed at evaluating the combined impacts of fire and vegetation on single soil characteristics and on the overall soil quality and functionality through two soil quality indices, simple additive (SQI) and a weighted function (SQI_FUNCT_). In order to reach the aims, burnt and unburnt soils were collected under different vegetation cover types (herbs and shrubs, black locust, pine and holm oak) within the Vesuvius National Park. The soils were analyzed for the main abiotic (water and organic matter content, total C, N, Ca, K, Cu and Pb concentrations, C/N ratio) and biotic (microbial and fungal biomasses, basal respiration, β-glucosidase activity) characteristics. On the basis of the investigated soil characteristics, several soil functions (water retention, nutrient supply, contamination content, microorganism habitat and activities), and the soil quality indices were calculated. The results showed that the impact of fire on soil quality and functionality was mediated by the vegetation cover type. In fact, fire occurrence led to a decrease in water and C/N ratio under herbs, a decrease in C concentration under holm oak and a decrease in Cu and Pb concentrations under pine. Although the soil characteristics showed significant changes according to vegetation cover types and fire occurrence, both the additive and weighted function soil quality indices did not significantly vary according to both fire occurrence and the vegetation cover type. Among the different vegetation cover types, pine was the most impacted one.

## 1. Introduction

Over the last decades, wildfires have burnt an average of about 4500 km^2^ in the Mediterranean regions of Europe. Wildfires were particularly severe in 2017, a year characterized by an intense heatwave coupled with a severe drought. Fires were particularly severe in southern Europe, causing extensive economic and ecological damages (i.e., biodiversity loss, carbon sequestration and raw material provisioning) and human casualties [1,2].

The dynamics of fire regimes in Southern Europe are driven by both natural and human factors, such as seasonal aridity, wind intensity, land cover heterogeneity, rural area abandonment, forest management practices and tourism [3]. In Mediterranean regions, the diversified landscapes and associated vegetation, ranging from trees (i.e., pines and oaks) to shrubs and herbs, particularly influence the fire regimes [4]. In addition, several invasive species have colonized Mediterranean regions [5], which represents a further element of complexity. In these diversified mosaics of vegetation, the impacts (in terms of spread and intensity) of fire are contrasting and difficult to predict, as they are mediated by the quantity and characteristics of plant fuel. In fact, plant species composition may influence the characteristics of fires, creating feedbacks that could lead to alternate woody-dominated and herb-dominated states [6].

Several studies have highlighted the impacts of fire on soil characteristics. The immediate and direct effects of fire are usually short-term and are restricted to the first few centimeters of the topsoil [7]. The indirect influence of fire in rangelands is the partial or total loss of plant and litter biomass, as well as changes in plant community composition and structure [8]. High temperatures, combustion of plant material, reactions of vaporization and condensation cause significant changes in important soil characteristics, modifying soil pH and nutrient cycles, altering the amount of organic matter, reducing the water availability, with consequences on soil organisms and activities [9,10,11]. In addition, the impacts of fires on soil characteristics can vary according to the vegetation cover type. For example, after fire occurrence, loss of nutrients is faster under herbs whereas soil water repellence is greater under tree species [12,13]. However, the combined effects of vegetation cover type and fire on soil characteristics and functions are poorly understood.

Although the effects of fire on a single soil factor have been widely studied, those on the overall soil quality are scarce, particularly in the Mediterranean area. Soil quality represents “the capacity of a soil to function, within ecosystem and land use boundaries, to sustain productivity, maintain environmental quality, and promote plant and animal health” [14,15]. Soil quality indices are useful as they integrate several soil characteristics into a single value and are more effective than a single characteristic in defining the soil disturbances.

With this framework, the research objectives were to: (i) evaluate the impact of fire and vegetation and their interaction on single soil; (ii) use a simple additive method (SQI) and a weighted additive method (SQI_FUNCT_) to evaluate the overall soil quality and functionality; (iii) highlight which among the studied vegetation cover types mitigated or enhanced the fire impact. Starting from a previous study [16], where no impacts of fire on soil quality index (SQI) were highlighted, the present research hypothesizes that soil quality indexes calculated by considering different functions (SQI_FUNCT_) could be more informative than the SQI. Moreover, the SQI_FUNCT_ could also highlight different fire impacts on the quality of soils under the studied vegetation cover types.

In order to achieve the aims, burnt and unburnt soils were collected under different vegetation cover types (herbs and shrubs, black locust, pine and holm oak) within the Vesuvius National Park. The soils were analyzed for the main abiotic characteristics and several soil functions.

## 2. Materials and Methods

### 2.1. Study Area

The study area is located at 12 km SE far from Naples, inside the Vesuvius National Park (Campania, Italy). The Vesuvius National Park, established in 1995, covers an area of 8482 ha and contains the original volcano, Mt. Somma (geographic coordinates: 40°50′14″ N–14°25′41″ E; maximum height: 1132 m a.s.l.) and Mt. Vesuvius (coordinates: 40°49′17″ N–14°25′32″ E; maximum height: 1281 m a.s.l.), originated from the 79 A.D. eruption. In June 2017, the Vesuvius National Park was affected by a severe fire [14] that caused the loss of more than 50% (approximately 3000 ha) of the existing plant cover [17]. Before the fire, the different areas inside the National Park were dominated by holm oaks (*Quercus ilex* L.), pines (*Pinus pinea* L., *Pinus nigra* L.), herbs and shrubs (such as *Myrtus communis* L., *Laurus nobilis* L., *Viburnum tinus* L., *Cistus* sp., *Ginesta* sp.) [18]. Additionally, few individuals of black locust (*Robinia pseudoacacia* L.), an invasive species, were randomly associated with the dominant species [5].

The Vesuvius mountain is characterized by Mediterranean climatic conditions with dry summers and rainy autumns and winters (mean annual temperature: 13.2 °C; annual precipitation: 960 mm, data from the reports of the Osservatorio Vesuviano).

### 2.2. Soil Sampling

The soils of the Vesuvius National Park show a silty-clay texture [19] and are classified as Lepti-Vitric Andosols [20]. The surface soils were sampled at 24 sites in June 2018, October 2018, February 2019 and May 2019 for a total of 96 soil samples. The sampling was performed within two consecutive days and after seven days without rainfall to minimize climatic variability. The 24 sites (approximately 400 m^2^ each) were equally divided into four different vegetation cover types: herbs and shrubs (H), black locust (BL), pines (P) and holm oak (HO). Within each vegetation cover type, three sites (affected by the same fire intensity) were collected in burnt (B) and three in unburnt (UB) areas. At each site, eight soil cores (0–10 cm deep, 0–5 cm diameter), after removing the ash and the thin layer of litter at the burnt sites and the deeper layer of litter at the unburnt sites, were collected and mixed together in order to obtain a homogeneous sample. The soil samples were put into sterile flasks and transported on ice to the laboratory [11,21].

### 2.3. Soil Chemical Analyses

In the laboratory, the soil samples were sieved (<2 mm) and analyzed for water and organic matter contents as well as for the total C and N contents. Soil water content (WC) was determined gravimetrically by drying fresh soil at 105 °C until reaching constant weight (approximately, 48–72 h); organic matter content (OM) was calculated multiplying by 1.724 the C_org_ [22] that was measured by Elemental analyzer (Thermo Finnigan, CNS Analyzer) on dried and pulverized (Fritsch Analysette Spartan 3 Pulverisette 0) samples (5 mg d.w.), previously saturated with HCl (10%, *v*:*v*); total carbon and nitrogen concentrations were determined by Elemental analyzer on dried and pulverized samples.

Total Ca, Cu, K and Pb concentrations were measured in oven-dried (75 °C) and pulverized soil samples, previously digested by hydrofluoric acid (50%) and nitric acid (65%) at a ratio of 1:2 (*v*:*v*) in a microwave oven (Milestone-Digestion/Drying Module mls 1200). The element concentrations in the digests were measured by Inductively Coupled Plasma Mass Spectrometry (ICP-MS Aurora M90, Bruker). The accuracy of Ca, Cu, K and Pb measurements was checked by concurrent analysis of standard reference material (BCR CRM 142R—Commission of the European Communities, 1994). The overall element recovery ranged from 80–120% for all the investigated soil samples.

All the above-described procedural steps were performed on three subsamples from each previously mixed sample.

### 2.4. Soil Biological Analyses

The microbial biomass (MB) was evaluated by SIR, the substrate-induced respiration method [23]. CO_2_ evolution from the soil after the addition of 2 mL of a D-glucose solution (75 mM) and incubation in sealed vials (30 mL) for 4 h at 25 °C in the darkness was measured through an infrared gas analyzer (Model LI6262, LI-COR, Lincoln, NE, USA). The fungal biomass (FB) was evaluated, after staining with aniline blue, through the membrane filter technique [24] determining hypha length with an optical microscope (Optika, B-252) by the intersection method [25].

Basal respiration (Resp) was estimated as CO_2_ evolution from the samples at 55% of water holding capacity after incubation in tight containers for 10 days at 25 °C by NaOH absorption followed by two-phase titration with HCl [26].

β-glucosidase activity (β-glu) was determined by adding 4 mL of modified universal buffer (MUB) pH 6 and 1 mL of 0.025 M p-nitrophenyl β-D-glucopyranoside (PNP) to 1 g of soil. The mixture was then incubated at 37 °C for 1 h, after which the enzymatic reaction was stopped by cooling on ice for 15 min. Then, 1 mL of 0.5 M CaCl_2_ and 4 mL of 0.1 M tris-hydroxymethilaminomethane-sodium hydroxide (THAM-NaOH) pH 12 was added. In the control, the substrate was added before the addition of CaCl_2_ and NaOH. The absorbance of the supernatant was measured at 420 nm and the results were expressed as mmol of PNP produced for 1 g of dry soil in 1 min [27,28]. The results were, respectively, expressed as mmol of fluorescein (FDA) and triphenylformazan (TPF) produced in 1 min for 1 g of dried soil.

Microbial and fungal biomasses, basal respiration and ♌-glucosidase activity were performed on soil samples stored at 4 °C within three days of the soil sampling. All the above-described procedural steps were performed on three subsamples from each previously mixed sample.

### 2.5. Soil Quality Index Calculations

All the SQI methods involved a set of 96 soil samples and of 13 soil characteristics considered as indicators of soil quality. The 13 indicators used for developing SQIs were soil water content, organic matter, total C and N contents, C/N ratio, total Ca, Cu, K and Pb concentrations, microbial and fungal biomasses, microbial respiration and ♌-glucosidase activity. Under the proposed framework, an ideal soil would have an SQI value of 1 for the highest quality soil and 0 for severely degraded soil. 

#### 2.5.1. Simple Additive SQI (SQI)

An integrated soil quality index was calculated taking into account the physico-chemical and biological characteristics that were ranked from 0 to 1, respectively reflecting low and high quality, according to Leitgib et al. [29]. The scores were assigned applying the *more is better* or *less is better* criteria, following this formula:

For *more is better*: x’ = (x − x_min_)/(x_max_ − x_min_)

For *less is better*: x’ = 1 − (x − x_min_)/(x_max_ − x_min_)

The *more is better* criterion was applied to water and organic matter contents, C and N concentrations, total nutrient concentrations (Ca and K), microbial and fungal biomasses, microbial respiration and ♌-glucosidase activity, for their roles in soil fertility, water partitioning and nutrient availability. On the contrary, the *less is better* criterion was applied to total trace metal (Cu and Pb) concentrations because their high concentration is potentially toxic for soil organisms, according to Marzaioli et al. [30]. Cu and Pb were chosen as markers of contamination as documented in previous research performed inside the Vesuvius National Park [18,31]. Regarding the C/N threshold value, the optimal range was identified between 9 and 11, thus scores were assigned by considering the *more is better* criterion for the values between 9 and 11 and the *less is better* criterion for the values <9 and >11.

For each site, the SQI was calculated, summing the parameter scores and dividing for the number of parameters, as reported by Andrews et al. [32]:

$$\mathrm{SQI}=\overset{n}{\underset{i=1}{\sum }}\frac{Si}{n}$$

where SQI is the soil quality index, S is the score assigned to each parameter and *n* is the number of the investigated parameters.

#### 2.5.2. SQI Calculated for Water Retention (SQI_WR_), Nutrient Supply (SQI_NS_), Contamination (SQI_C_), Microorganism Habitat (SQI_MH_) and Microorganism Activity (SQI_MA_) Functions

In this approach, the aforementioned 13 soil characteristics, scored 0 to 1 as above described, were grouped into the following five categories (Table 1) that express crucial soil functions: water retention (WR), nutrient supply (NS), contamination (C), microorganism habitat (MH) and activity (MA). The SQIs of functions were ranked from 0 to 1, respectively reflecting low and high quality. Within each function, the soil characteristics were weighted and their values were summed up to 1 [33]. The value of each function was expressed as the arithmetic average of the values of the descriptors included in that category. Use of the arithmetic average assumes equal importance of each characteristic in the soil [34].

#### 2.5.3. SQI Calculated as Weighted Functions (SQI_FUNCT_)

The SQIs calculated for each function were integrated into a single index (SQI_FUNCT_) by calculating their arithmetic average, which assumes equal importance of each function [34]. The SQI_FUNCT_ was calculated as follows:

SQI_FUNCT_ = (0.2 × SQI_WR_) + (0.2 × SQI_NS_) + (0.2 × SQI_C_) + (0.2 × SQI_MH_) + (0.2 × SQI_MA_)

### 2.6. Statistical Analyses

As the investigated soil characteristics and the SQI did not match the basic assumptions of normality and homoscedasticity required for parametric statistics, the Wilk–Shapiro test (for α = 0.05; *n* = 96) and the Wilcoxon test (for α = 0.05; *n* = 96) were performed to evaluate the differences in each investigated soil characteristics and SQIs between burnt (B) and unburnt (UB) sites, and the Kruskal–Wallis Rank-Sum test (for α = 0.05; *n* = 96) with Bonferonni adjustment was performed to compare the differences in each investigated soil characteristic and SQI among the different vegetation cover types.

In order to exclude the influence of the sampling time and to highlight the direct influences of vegetation and fire and their interactions on soil characteristics (WC, OM, total C and N concentrations, C/N, total Ca, K, Cu and Pb concentrations, MB, FB, Resp, β-glu activity) and on soil quality indices and functions (SQI, SQI_WR_, SQI_NS_, SQI_CC_, SQI_MH_, SQI_MA_ and SQI_FUNCT_) linear mixed effect models (LME) were used. For each soil characteristic and index, the influence of vegetation and fire, considered as fixed effects, and of sampling time, considered as a random effect, was calculated using restricted maximum likelihood (REML) for better estimation of the variance components for the present dataset. The significant impacts and interactions among vegetation, fire and sampling time on soil characteristics and indices were calculated with the comparison of models, using the likelihood ratio test with the Anova function (for a = 0.05).

The R 3.6.2 programming environment (R Development Core Team) was used to perform the statistical analyses and the linear mixed effect models (lme4 package). The graphs were created by SigmaPlot12 software (Jandel Scientific, San Rafael, CA, USA).

## 3. Results

### 3.1. Soil Abiotic and Biotic Characteristics

At unburnt sites, the soil characteristics ranged widely among different vegetation cover types. In particular, water content and C concentrations, respectively, ranged from 9 to 35% d.w. and from 2.6 to 9.5% d.w. with values significantly higher in soils under holm oak (Table 2). The organic matter content and N concentrations, respectively, ranged from 3 to 12% d.w. and from 0.2 to 0.7% d.w. with values significantly higher in soils under holm oak and black locust (Table 2). Soil C/N ratios ranged from 10 to 15 and did not show any significant differences among the vegetation cover types (Table 2). Total Ca, K, Cu and Pb concentrations slightly varied in soils, and no significant differences occurred among the vegetation cover types (Table 2). Microbial and fungal biomasses, respectively, ranged from 0.9 to 2.1 mg C g^−1^ d.w. and from 0.4 to 1.2 mg g^−1^ d.w. with values significantly higher in soil under holm oak (Table 3). The basal respiration ranged from 1.7 to 6.7 mg CO_2_ g^−1^ d.w. and ♌-glucosidase activity ranged from 4.8 to 11 mmol PNP min^−1^ g^−1^ d.w., with values significantly higher in soils under herbs and holm oak, respectively (Table 3).

At burnt sites, water content ranged from 5.3 to 25% d.w. with values significantly higher in soils under holm oak (Table 2). The organic matter content and C concentrations, respectively, ranged from 5 to 10% d.w. and from 1.2 to 5.6% d.w. with values significantly higher in soils under holm oak and black locust (Table 2). N concentrations and C/N ratios, respectively, ranged from 0.1 to 0.7% d.w. and from 8 to 16 with values significantly higher in soils under black locust and pine, respectively (Table 2). Total Ca and K concentrations, respectively, ranged from 51 to 69 mg g^−1^ d.w. and from 24 to 36 mg g^−1^ d.w. and did not significantly vary among the vegetation cover types (Table 2). Total Cu and Pb concentrations, respectively, ranged from 0.2 to 0.11 mg g^−1^ d.w. and from 0.1 to 0.6 mg g^−1^ d.w. with values significantly lower under pine (Table 2). The microbial biomass ranged from 0.5 to 1.8 mg C g^−1^ d.w. with values significantly higher under holm oak and pine (Table 3) whereas the fungal biomass ranged from 0.3 to 1.2 mg g^−1^ d.w. with values significantly higher under holm oak (Table 3). The basal respiration ranged from 1.2 to 3.3 mg CO_2_ g^−1^ d.w. and did not significantly vary among the vegetation cover types (Table 3). Finally, ♌-glucosidase activity ranged from 3 to 8.5 mmol PNP min^−1^ g^−1^ d.w. with values statistically higher under holm oak (Table 3).

The comparison of each soil characteristic under the same vegetation cover type between unburnt and burnt sites demonstrated the following: (1) water content and C/N ratios were significantly higher in unburnt soils under herbs, (2) C concentrations were significantly higher in unburnt soils under holm oak and (3) total Cu and Pb concentrations were significantly higher in unburnt soils under pine (Table 2).

The linear mixed-effect model showed that sampling time (random effects) had no significant influence on any soil characteristics whereas vegetation cover type and fire occurrence and, to a lesser extent, their interactions (fixed effects) influenced soil indicators (Table 4). In particular, OM content, microbial and fungal biomasses, respiration and ♌-glu activity (Table 4) were affected by vegetation cover types; Cu and Pb concentrations were affected by fire occurrence; WC, C, Ca and K concentrations were affected by both vegetation cover type and fire occurrence and by their interaction (Table 4); finally, C/N ratios were affected by the interaction between vegetation cover type and fire occurrence (Table 4).

### 3.2. Soil Quality Indices

**Figure 1:**
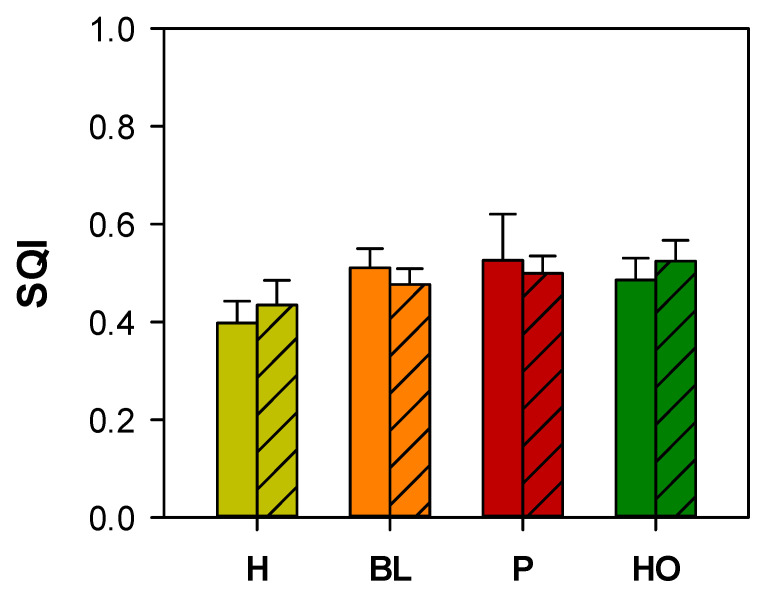
Mean values (±st. err.) of soil quality index (SQI) calculated in unburnt (no pattern) and burnt (coarse pattern) soil samples collected under different vegetation cover types (herbs—H, light green; black locust—BL, orange; pine—P, red; holm oak—HO, dark green). No significant differences among vegetation cover types and between burnt and unburnt soils were detected (Kruskal–Wallis test).

The Soil Quality Index (SQI), calculated taking into account all the soil characteristics, ranged from 0.4 to 0.5 in unburnt soils and from 0.4 to 0.6 in burnt sites and did not show significant differences among the vegetation cover types (Figure 1). Moreover, the SQI did not show significant differences between unburnt and burnt sites with the same vegetation cover type (Figure 1).

**Figure 2:**
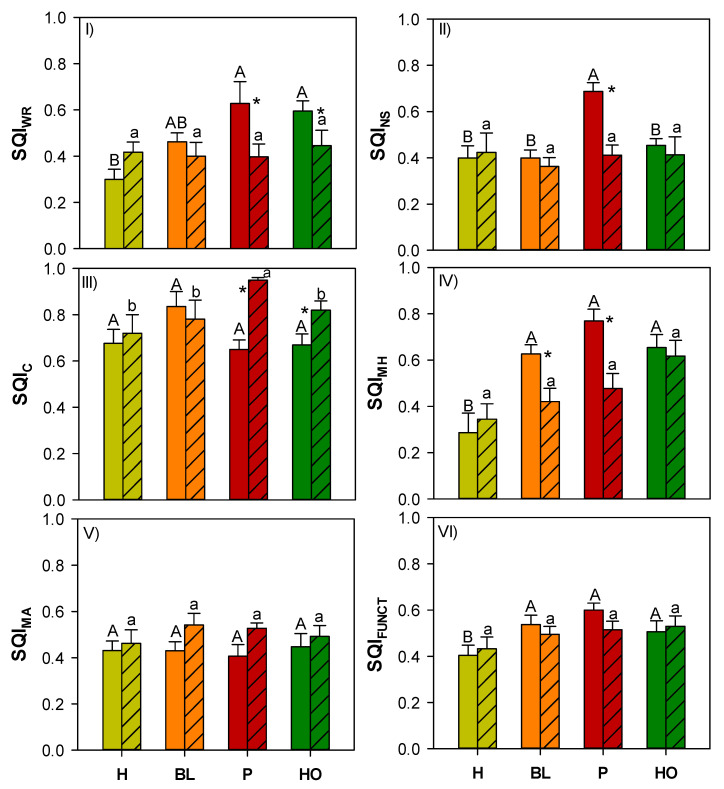
Mean values (±st. err.) of SQIs calculated for each function (water retention—SQI_WR_, nutrient supply—SQI_NS_, contamination—SQI_C_, microorganism habitat—SQI_MH_ and microorganism activity—SQI_MA_), and on the basis of all functions (SQI_FUNCT_) calculated in unburnt (no pattern) and burnt (coarse pattern) soils samples under different vegetation cover type (herbs—H, light green; black locust—BL, orange; pine—P, red; holm oak—HO, dark green). Different capital and small letters indicate significant differences (at least, *p* < 0.05, Kruskal–Wallis test) in soil characteristics among vegetation cover types, respectively, at unburnt and burnt sites. (Kruskal–Wallis test). Asterisks indicate significant differences (*p* < 0.05) between unburnt and burnt sites within the same vegetation cover type (Wilcoxon test).

At unburnt sites, water retention SQI (SQI_WR_) ranged from 0.3 to 0.6 with values significantly higher under pine and holm oak (Figure 2I). The nutrient supply SQI (SQI_NS_) ranged from 0.4 to 0.7 and was significantly higher under pine (Figure 2II). The contamination SQI (SQI_C_) ranged from 0.6 to 0.8 (Figure 2III) and the microorganism activity SQI (SQI_MA_) was approximately 0.4 (Figure 2IV) and did not significantly vary among vegetation cover types. The microorganism habitat SQI (SQI_MH_) ranged from 0.3 to 0.8, with values significantly higher under pine and black locust (Figure 2V). The weighted function SQI (SQI_FUNCT_) ranged from 0.4 to 0.6 with lower values under herbs (Figure 2VI).

In burnt sites, SQI_WR_ and SQI_NS_ showed values of approximately 0.4, SQI_MA_ showed values of approximately 0.5 and SQI_MH_ ranged from 0.3 to 0.6; these indexes did not significantly vary according to vegetation cover types (Figure 2I,II,IV,V). The SQI_C_ ranged from 0.7 to 0.9 with values significantly higher in soil covered by pine and holm oak (Figure 2III). Finally, the SQI_FUNCT_ ranged from 0.4 to 0.5 and did not significantly vary according to the vegetation cover types (Figure 2VI).

The comparison of the different soil quality and function indices at unburnt and burnt sites covered by the same vegetation highlighted that SQI_WR_ and SQI_C_ were significantly higher under pine and holm oak in unburnt soil (Figure 2I,III), SQI_NS_ showed significantly higher value under pine in unburnt soil (Figure 2II) and SQI_MH_ showed higher values under black locust and pine in unburnt soil (Figure 2IV).

The linear mixed-effect model showed that soil quality indices (SQIs) were mainly dependent on vegetation cover type, fire and their interactions (fixed effects) and they were not dependent on sampling time (random effects) (Table 5). In particular, SQI, SQI_WR,_ SQI_C_, SQI_MH,_ SQI_FUNCT_ were affected by vegetation cover types (Table 5); SQI_C_ and SQI_MA_ were affected by fire (Table 5) whereas SQI_WR_, SQI_NS_, SQI_MH_ and SQI_CC_ were affected by the interactions between vegetation cover types and fire occurrence (Table 5).

## 4. Discussion

In the investigated area, vegetation cover type had the largest and most consistent influence on soil characteristics and functions. Within a vegetation cover type, there were few significant differences between burnt and unburnt soils. Then, in the unburnt study area, soil quality and functionality mainly depended on vegetation cover types. In particular, the presence of trees positively affected water and organic matter contents, C and N concentrations, microbial and fungal biomasses, β-glu activity were significantly higher under holm oak, pine and, to a lesser extent, under black locust than under herbs. The highest soil organic matter content, responsible for high water retention [35] under trees, could be a result of a large amount of litterfall, deriving from the high plant productivity [12]. Moreover, the highest soil N amount could be due to different plant strategies. In fact, thanks to its deep rooting system, holm oak contributes to bringing up nutrients from lower soil layers [36], that are retained thanks to either clays or organic matter content whereas black locust enriches N soil amount thanks to symbiotic root association with N fixator organisms [37]. The aboveground net primary production and the nutrient content under tree vegetation led to an increase in microbial biomass and enzymatic activity in the investigated area. These characteristics contribute to creating specific relationships between the dominant plant species and the soil microbial component [38]. Despite the high microorganism abundance, the soils covered by holm oak and pine showed low basal respiration, probably due to the to the abundant inhibitory compounds of sclerophyll Mediterranean species, that have been shown to slow down decomposition and mineralization by forming recalcitrant complexes with organic matter [39]. The great litter amount observed under trees likely was responsible for the wide water retention and the great variability in microorganism niches that, in turn, increased the SQI_WR_, SQI_NS_ and SQI_MH_. These results highlight the positive effects of tree species on the soil functionality of the Mediterranean environment, as it has been shown that dense plant cover limits nutrient loss, erosion and fast organic matter degradation [40].

In the investigated area, fire impact was mediated by the dominant cover type, similar results were reported by Wragg et al. [6] for burnt and unburnt temperate grassland soils. Fire effects were particularly pronounced in soil covered by herbs, where water content and C/N significantly decreased as compared to the unburnt soil. At the burnt sites covered by herbs, almost all the vegetation was destroyed, contributing to increased soil water evaporation and carbon compound volatilization [11]. Moreover, fire also affected C in soils covered by holm oak and Cu and Pb in soils covered by pine. The destruction of the abundant litter layer under holm oak could have contributed to the strong reduction of C in holm oak burnt sites. Whereas the decrease in soil Cu and Pb concentrations under burnt pine was probably due to brief-term leaching phenomena [41,42]. Although the greater decrease in soil water content was measured in soils covered by herbs, fire occurrence negatively impacted the water retention function (SQI_WR_) under pine and holm oak as well, probably because of the destruction of the litter layer strongly involved in soil humidity retention [35], which is greater under trees than under shrubs and herbs. After fire, soils under pine also lost part of the nutrient supply function (SQI_NS_) due to the widespread leaching or erosion phenomena occurring in the early post-fire succession stage [43]. For similar reasons, the same phenomenon seemed responsible for the decrease in soil contamination degree, as Cu and Pb concentrations decreased, causing the increase in the SQI_C_ under pine and holm oak. By contrast, in burnt soils covered by pine and black locust, the microorganism habitat function decreased, especially for fungal biomass as compared to microbial biomass. These findings confirm the negative impact of fire on soil microorganisms. Burning of the organic layer and heat transfer to the soil during fires leads to microbial mortality immediately after the fire [44]. However, the slight microbial biomass increase observed in burnt soil suggests that microbes quickly recover, due to the successful colonization and survival of many phyla into the soil during the process of post-fire succession [45].

Although the soil characteristics and functions showed significant changes according to vegetation cover type and fire occurrence, both the additive (SQI) and weighted function (SQI_FUNCT_) soil quality indices showed a medium soil quality and did not significantly vary according to the vegetation cover type. The results obtained by the SQI agree with Memoli et al. [16] and could be due to the fact that the fire responses of the single characteristics sometimes were opposite, suggesting that they can be compensated when their interactions are considered. However, the integrated SQI is dependent on several factors such as the design of the study, choice of soil parameters included in the model to compute SQI and endpoint variables [46]. Similarly, the weighted function soil quality index (SQI_FUNCT_) also did not vary among vegetation cover type and fire occurrence, but their values were slightly higher than SQI in all the treatments. This result indicated that the soil quality can be predicted from appropriate weightage on soil functions (SQI_FUNCT_) in accordance with several studies [46,47,48,49]. In fact, the evaluation of the overall soil quality in response to fire could be difficult as the fire exerts opposite responses on different soil characteristics and, in turn, on functions.

## 5. Conclusions

In the investigated area, the impact of fire on soil quality and functionality was mediated by the vegetation cover type. In particular, fire occurrence caused a significant decrease in water and C/N ratio under herbs, a significant decrease in C concentration under holm oak and a decrease in Cu and Pb concentrations under pine. Soil functions were also impacted both by fire and vegetation cover type, with a decrease in water retention under pine and holm oak, a decrease in nutrient supply under pine, an increase in contamination quality under pine and holm oak and a decrease in microorganism habitat under pine and black locust.

Although the soil indicators showed significant changes according to vegetation cover types and fire occurrence, both the additive (SQI) and weighted function (SQI_FUNCT_) soil quality indices did not significantly vary according to both fire occurrence and the vegetation cover type. This could suggest that the overall soil quality was slightly impacted by fire, as soil indicators and functions showed opposite responses which masked the overall effect on the whole soil quality.

Soils under pine were the most fire-impacted, as variations in water retention, nutrient supply, contamination and microorganism habitat were of greater extent as compared to those under the other vegetation cover types; by contrast, soils under herbs seemed to be less impacted by fire, as no differences in soil functions were observed between burnt and unburnt soils.

The simultaneous investigation of burnt and unburnt soils under the investigated vegetation cover types could be informative for fire management in order to prevent and mitigate fire impacts on soil and to restore burnt areas in the Mediterranean environment.

## Figures and Tables

**Table 1:** Weights assigned to each investigated soil characteristic grouped in the five considered soil functions.

Soil Function	Soil Characteristics	Weight (A)
Water retention	Water content	0.5
	Organic matter content	0.5
Nutrient Supply	Total C	0.25
	Total N	0.25
	Total Ca	0.25
	Total K	0.25
Contamination	Total Cu	0.5
	Total Pb	0.5
Microorganism habitat	Microbial biomass	0.5
	Fungal biomass	0.5
Microorganism activity	Respiration	0.33
	♌-glu activity	0.33
	C/N	0.34

**Table 2:** Mean values (±st. err.) of water (WC, expresses as % d.w.) and organic matter contents (OM, expresses as % d.w.), C and N concentrations (expressed as % d.w.), C/N ratios, total concentrations of Ca, K, Cu and Pb (expressed as mg g^−1^ d.w.) measured under different vegetation cover types (herbs, Black locust, pine and Holm oak) in unburnt (UB) and burnt (B) soils collected at the Vesuvius National Park.

Vegetation Cover Type	Fire	WC	OM	C	N	C/N	Ca	K	Cu	Pb
**Herbs**	**UB**	11.9 B (±3.74)	3.08 B (±0.94)	2.79 B (±0.97)	0.27 B (±0.07)	14.9 A (±2.23)	57.9 A (±12.1)	31.7 A (±5.06)	0.07 A (±0.01)	0.05 A (±0.01)
	**B**	5.34 c * (±1.35)	4.99 b (±1.61)	1.24 c (±0.48)	0.14 b (±0.02)	7.89 b * (±1.89)	52.6 a (±12.5)	36.1 a (±8.14)	0.11 a (±0.03)	0.05 a (±0.01)
**Black locust**	**UB**	10.0 B (±2.23)	8.12 A (±1.44)	3.35 B (±0.64)	0.67 A (±0.34)	9.98 A (±1.68)	59.6 A (±12.5)	28.2 A (±4.75)	0.11 A (±0.03)	0.05 A (±0.01)
	**B**	8.96 bc (±1.73)	8.30 a (±1.32)	5.43 a (±1.34)	0.73 a (±0.24)	9.31 b (±1.14)	51.3 a (±10.9)	28.3 a (±3.80)	0.08 a (±0.01)	0.04 a (±0.01)
**Pine**	**UB**	11.5 B (±2.20)	5.27 B (±0.80)	2.61 B (±0.26)	0.21 B (±0.01)	12.4 A (±1.07)	63.9 A (±18.1)	27.6 A (±7.03)	0.08 A * (±0.02)	0.04 A * (±0.01)
	**B**	11.2 b (±1.61)	5.30 b (±0.63)	3.08 b (±0.26)	0.19 b (±0.02)	16.4 a (±2.55)	61.9 a (±11.0)	24.5 a (±3.24)	0.02 b (<0.01)	0.01 b (<0.01)
**Holm oak**	**UB**	34.8 A (±4.36)	12.1 A (±1.86)	9.47 A (±1.31)	0.61 A (±0.09)	12.6 A (±1.42)	57.9 A (±10.8)	30.5 A (±3.79)	0.07 A (±0.01)	0.07 A (±0.01)
	**B**	25.1 a (±4.12)	10.4 a (±2.11)	5.69 a * (±1.12)	0.39 ab (±0.04)	12.1 ab (±2.70)	69.3 a (±16.6)	30.4 a (±5.65)	0.07 a (±0.02)	0.06 a (±0.01)

* *p* < 0.5. Different capital and small letters indicate significant differences (at least, *p* < 0.05, Kruskal–Wallis test) in soil characteristics among vegetation cover types, respectively, at unburnt and burnt sites. Asterisks indicate statistically significant differences (at least, *p* < 0.05, Wilcoxon test) in soil characteristics between unburnt and burnt sites covered by the same vegetation cover type.

**Table 3:** Mean values (±st. err.) of microbial biomass (MB, expressed as mg C g^−1^ d.w.), fungal biomass (FB, expressed as mg g^−1^ d.w.), basal respiration (Resp, expressed as mg CO_2_ g^−1^ d.w.) and ♌-glucosidase activity (β-glu, expressed as mmol PNP min^−1^ g^−1^ d.w.) measured under different vegetation cover types (herbs, Black locust, pine and Holm oak) in unburnt (UB) and burnt (B) soils collected at the Vesuvius.

Vegetation	Fire	MB	FB	Resp	♌-glu
**Herbs**	**UB**	0.92 B (±0.30)	0.35 B (±0.06)	6.67 A (±2.26)	4.75 B (±1.42)
	**B**	0.53 b (±0.10)	0.26 b (±0.06)	3.32 a (±0.55)	3.01 c (±0.46)
**Black locust**	**UB**	1.27 AB (±0.28)	0.53 AB (±0.13)	2.48 B (±0.69)	7.72 AB (±1.47)
	**B**	1.39 ab (±0.27)	0.49 ab (±0.09)	1.75 a (±0.43)	6.26 ab (±1.08)
**Pine**	**UB**	0.88 B (±0.31)	0.55 AB (±0.21)	1.78 B (±0.69)	4.78 B (±1.49)
	**B**	1.55 a (±0.19)	0.66 ab (±0.11)	1.22 a (±0.26)	5.35 b (±0.46)
**Holm oak**	**UB**	2.12 A (±0.26)	1.15 A (±0.24)	1.68 B (±0.48)	10.6 A (±1.86)
	**B**	1.83 a (±0.27)	1.15 a (±0.32)	1.57 a (±0.36)	8.43 a (±1.15)

National Park. Different capital and small letters indicate significant differences (at least, *p* < 0.05, Kruskal–Wallis test) in soil characteristics among vegetation cover types, respectively, at unburnt and burnt sites.

**Table 4:** Summary of mixed-effect model analyses (F-value: *F*) between vegetation cover type (Veg) and fire (Fire) as fixed effects and sampling time as random effect, on the indicators (water content—WC, organic matter content—OM, C, N concentration, C/N ratio, Ca, K, Cu and Pb concentrations, microbial biomass—MB, fungal biomass—FB, basal respiration—Resp, β-glucosidase activity—β-glu) of soils collected at the Vesuvius National Park.

		Fixed Effects		Random Effect	Interactions between Fixed Factors
		Veg	Fire	Sampling Time	Veg × Fire
**WC**	*F*	6.85 *	1.85 *	0.18	1.72 *
**OM**	*F*	0.18 *	1.23	0.50	0.23
**C**	*F*	2.49 *	2.36 *	0.64	0.51 *
**N**	*F*	1.50	0.85	1.19	0.33
**C/N**	*F*	0.56	0.26	1.31	1.38 *
**Ca_tot_**	*F*	12.5 *	<0.01 *	0.26	0.86 ***
**K_tot_**	*F*	61.8 *	<0.01 ***	9.87	5.00 ***
**Cu_tot_**	*F*	4.73	1.57 *	0.31	1.31
**Pb_tot_**	*F*	2.52	1.26 *	0.86	0.71
**MB**	*F*	1.10 *	0.18	1.41	0.29
**FB**	*F*	5.59 ***	0.16	2.21	0.37
**Resp**	*F*	4.54 **	10.8	0.82	2.00
**B-glu**	*F*	0.34 *	2.93	0.28	0.80

* *p* < 0.5, ** *p* < 0.01, *** *p* < 0.001. Asterisks indicate significant impacts of fixed effects and their interactions on soil characteristics (Anova test—model comparison).

**Table 5:** Summary of mixed-effect model analyses (F-value: *F*) between vegetation cover type (Veg) and fire (Fire) as fixed effects and sampling time as random effect, on soil quality indices (simple additive soil quality indices: SQI, water retention soil quality index—SQI_WR_, nutrient supply soil quality index—SQI_NS_, contamination soil quality index—SQI_C_, microorganism habitat soil quality index—SQI_MH_, microorganism activity soil quality index—SQI_MA_, weighted function soil quality index—SQI_FUNCT_) calculated on soils collected at the Vesuvius National Park.

		Fixed Effects		Random Effect	Interactions between Fixed Factors
		Veg	Fire	Sampling Time	Veg × Fire
**SQI**	*F*	3.29 *	0.70	16.2	0.63
**SQI_WR_**	*F*	3.06 *	1.62	7.21	0.90 *
**SQI_NS_**	*F*	1.64	0.11	14.2	3.39 *
**SQI_C_**	*F*	4.94 **	2.34 *	43.0	3.25 *
**SQI_MH_**	*F*	8.59 ***	1.16	4.19	2.04 *
**SQI_MA_**	*F*	0.48	5.54 *	8.63	0.21
**SQI_FUNCT_**	*F*	4.49 **	0.27	15.4	0.73

* *p* < 0.5, ** *p* < 0.01, *** *p* < 0.001. Asterisks indicate significant impacts of fixed effects and their interactions on soil characteristics (Anova test—model comparison).

## Data Availability

The data that has been used is confidential.

## References

[1] Turco M., von Hardenberg J., AghaKouchak A., Llasat M.C., Provenzale A., Trigo R.M. (2017). On the key role of droughts in the dynamics of summer fires in Mediterranean Europe. Sci. Rep..

[2] Turco M., Rosa-Cánovas J.R., Bedia J., Jerez S., Montávez J.P., Llasat M.C., Provenzale A. (2018). Exacerbated fires in Mediterranean Europe due to anthropogenic warming projected with nonstationary climate-fire models. Nat. Commun..

[3] San-Miguel-Ayanz J., Schulte E., Schmuck G., Camia A., Tiefenbacher J. (2012). Comprehensive monitoring of wildfires in Europe: The European forest fire information system (EFFIS). Approaches to Managing Disaster-Assessing Hazards, Emergencies and Disaster Impacts.

[4] Medail F. (2017). The specific vulnerability of plant biodiversity and vegetation on Mediterranean islands in the face of global change. Reg. Environ. Chang..

[5] De Marco A., Esposito F., Berg B., Giordano M., Virzo De Santo A. (2013). Soil C and N sequestration in organic and mineral layers of two coeval forest stands implanted on pyroclastic material (Mount Vesuvius, South Italy). Geoderma.

[6] Wragg P.D., Mielke T., Tilma D. (2018). Forbs grasses, and grassland fire behaviour. J. Ecol..

[7] Girona-García A., Ortiz-Perpiñá O., Badía-Villas D. (2019). Dynamics of topsoil carbon stocks after prescribed burning for pasture restoration in shrublands of the Central Pyrenees (NE-Spain). J. Environ. Manag..

[8] Halofsky J.E., Peterson D.L., Harvey B.J. (2020). Changing wildfire, changing forests: The effects of climate change on fire regimes and vegetation in the Pacific Northwest, USA. Fire Ecol..

[9] Quigley K.M., Wildt R.E., Sturtevant R.B., Kolka R.K., Dickinson M.B., Kern C.C., Donner D.M., Miesel J.R. (2019). Fuels, vegetation, and prescribed fire dynamics influence ash production and characteristics in a diverse landscape under active pine barrens restoration. Fire Ecol..

[10] Fattorini S. (2010). Effects of fire on tenebrionid communities of a *Pinus pinea* plantation: A case study in a Mediterranean site. Biodivers. Conserv..

[11] Santorufo L., Memoli V., Panico S.C., Santini G., Barile R., Di Natale G., Trifuoggi M., De Marco A., Maisto G. (2021). Early post-fire changes in properties of Andosols within a Mediterranean area. Geoderma.

[12] De Marco A., Meola A., Esposito F., Virzo De Santo A. (2008). Productivity and modifications of ecosystem processes in gaps of a low Macchia in southern Italy. Web Ecol..

[13] Panico S.C., Ceccherini M.T., Memoli V., Maisto G., Pietramellara G., Barile R., De Marco A. (2020). Effects of different vegetation types on burnt soil properties and microbial communities. Int. J. Wildland Fire.

[14] Doran J.W., Parkin T.B., Doran J.W., Coleman D.C., Bezdicek D.F., Stewart B.A. (1994). Defining and assessing soil quality. Defining Soil Quality for a Sustainable Environment.

[15] Doran J.W., Parkin T.B., Doran J.W., Jones A.J. (1996). Quantitative indicators of soil quality: A minimum data set. Methods for Assessing Soil Quality.

[16] Memoli V., Panico S.C., Santorufo L., Barile R., Di Natale G., Di Nunzio A., Toscanesi M., Trifuoggi M., De Marco A., Maisto G. (2020). Do Wildfires Cause Changes in Soil Quality in the Short Term?. Int. J. Environ. Res. Public Health.

[17] Saulino L., Rita A., Migliozzi A., Maffei C., Allevato E., Garonna A.P., Saracino A. (2020). Detecting burn severity across Mediterranean forest types by coupling medium-spatial resolution satellite imagery and field data. Remote Sens..

[18] Memoli V., Eymar E., García-Delgado C., Esposito F., Santorufo L., De Marco A., Barile R., Maisto G. (2018). Total and fraction content of elements in volcanic soil: Natural or anthropogenic derivation. Sci. Total Environ..

[19] Santorufo L., Cortet J., Nahmani J., Pernin C., Salmon S., Pernot A., Morel J.L., Maisto G. (2015). Responses of functional and taxonomic collembolan community structure to site management in Mediterranean urban and surrounding areas. Eur. J. Soil Biol..

[20] Di Gennaro A. (2002). I Sistemi di Terre Della Campania.

[21] Panico S.C., Memoli V., Santorufo L., Esposito F., De Marco A., Barile R., Maisto G. (2021). Linkage between Site Features and Soil Characteristics within a Mediterranean Volcanic Area. Front. For. Glob. Chang..

[22] Pribyl D.W. (2010). A critical review of the conventional SOC to SOM conversion factor. Geoderma.

[23] Degens B.P., Schipper L.A., Sparling G.P., Vojvodic-Vukovic M. (2000). Decreases in organic C reserves in soils can reduce the catabolic diversity of soil microbial communities. Soil Biol. Biochem..

[24] Sundman V., Sivela S. (1978). A comment on the membrane filter technique for the estimation of length of fungal hyphae in soil. Soil Biol. Biochem..

[25] Olson F.C.W. (1950). Quantitative estimates of filamentous algae. Trans. Am. Microsc. Soc..

[26] Anderson T.H., Domsch K.H. (1978). A physiological method for the quantitative measurements of microbial biomass in soil. Soil Biol. Biochem..

[27] Tabatabai M.A., Page A.L., Miller R.H., Keeney D.R. (1982). Soil Enzymes. Methods of Soil Analysis.

[28] Tabatabai M.A., Bremner J.M. (1969). Use of p-nitrophenyl phosphate for assay of soil phosphatase activity. Soil Biol. Biochem..

[29] Leitgib L., Kálmán J., Gruiz K. (2007). Comparison of bioassays by testing whole soil and their water extract from contaminated sites. Chemosphere.

[30] Marzaioli R., D’Ascoli R., De Pascale R.A., Rutigliano F.A. (2010). Soil quality in a Mediterranean area of Southern Italy as related to differentland use types. Appl. Soil Ecol..

[31] Memoli V., Esposito F., Panico S.C., De Marco A., Barile R., Maisto G. (2019). Evaluation of tourism impact on soil metal accumulation through single and integrated indices. Sci. Total Environ..

[32] Andrews S.S., Karlen D.L., Cambardella C.A. (2004). The soil management assessment framework: A quantitative soil quality evaluation method. Soil Sci. Soc. Am. J..

[33] Fernandes J.C., Gamero C.A., Rodrigues J.G.L., Miràs-Avalos J.M. (2011). Determination of the quality index of a Paleudult under sunflower culture and different management systems. Soil Till. Res..

[34] Lima A.C.R., Brussaard L., Totola M.R., Hoogmoed W.B., de Goede R.G.M. (2013). A functional evaluation of three indicator sets for assessing soil quality. Appl. Soil Ecol..

[35] Yang F., Zhang G.L., Yang J.L., Li D.C., Zhao Y.G., Liu F., Yang R.M., Yang F. (2014). Organic matter controls of soil water retention in an alpine grassland and its significance for hydrological processes. J. Hydrol..

[36] Moreno G., Obrador J.J. (2007). Effects of trees and understorey management on soil fertility and nutritional status of holm oaks in Spanish dehesas. Nutr. Cycl. Agroecosyst..

[37] Rice S.K., Westerman B., Federici R. (2004). Impacts of the exotic, nitrogen-fixing black locust (*Robinia pseudoacacia*) on nitrogen-cycling in a pine–oak ecosystem. Plant Ecol..

[38] Steinauer K., Tilman D., Wragg P.D., Cesarz S., Cowles J.M., Pritsch K., Reich P.B., Weisser W.W., Eisenhauer N. (2015). Plant diversity effects on soil microbial functions and enzymes are stronger than warming in a grassland experiment. Ecology.

[39] Incerti G., Bonanomi G., Giannino F., Rutigliano F.A., Piermatteo D., Castaldi S., De Marco A., Fierro A., Fioretto A., Maggi O. (2011). Litter decomposition in Mediterranean ecosystems: Modelling the controlling role of climatic conditions and litter quality. Appl. Soil Ecol..

[40] Mancini Teixeira H., Cardoso I.M., Bianchi F.J.J.A., da Cruz Silva A., Jamme D., Peña-Claros M. (2020). Linking vegetation and soil functions during secondary forest succession in the Atlantic forest. For. Ecol. Manag..

[41] Certini G. (2005). Effects of fire on properties of forest soils: A review. Oecologia.

[42] Francos M., Úbeda X., Pereira P. (2019). Impact of torrential rainfall and salvage logging on post-wildfire soil properties in NE Iberian Peninsula. Catena.

[43] Kong J., Yang J., Bai E. (2018). Long-term effects of wildfire on available soil nutrient composition and stoichiometry in a Chinese boreal forest. Sci. Total Environ..

[44] Dooley S.R., Treseder K.K. (2012). The effect of fire on microbial biomass: A meta-analysis of field studies. Biogeochemistry.

[45] Xiang X., Shi Y., Yang J., Kong J., Lin X., Zhang H., Zeng J., Chu H. (2012). Rapid recovery of soil bacterial communities after wildfire in a Chinese boreal forest. Sci. Rep..

[46] Mukherjee A., Lal R. (2014). Comparison of Soil Quality Index Using Three Methods. PLoS ONE.

[47] AbdelRahman M.A.E., Shalaby A., Mohamed E.S. (2019). Comparison of two soil quality indices using two methods based on geographic information system. Egypt. J. Remote Sens. Space Sci..

[48] Bouma J. (2014). Soil science contributions towards sustainable development goals and their implementation: Linking soil functions with ecosystem services. J. Plant Nutr. Soil Sci..

[49] Baveye P.C., Baveye J., Gowdy J. (2016). Soil “Ecosystem” Services and Natural Capital: Critical Appraisal of Research on Uncertain Ground. Front. Environ. Sci..

